# Association of body composition with neuroimaging biomarkers and cognitive function; a population-based study of 70-year-olds

**DOI:** 10.1016/j.ebiom.2024.105555

**Published:** 2025-01-08

**Authors:** Jessica Samuelsson, Anna Marseglia, Ola Wallengren, Olof Lindberg, Caroline Dartora, Nira Cedres, Sara Shams, Silke Kern, Anna Zettergren, Eric Westman, Ingmar Skoog

**Affiliations:** a Neuropsychiatric Epidemiology Unit, Department of Psychiatry and Neurochemistry, Institute of Neuroscience and Physiology, Sahlgrenska Academy, Centre for Ageing and Health (AGECAP) at the University of Gothenburg, Sweden; b Division of Clinical Geriatrics, Center for Alzheimer Research, Department of Neurobiology, Care Sciences and Society, Karolinska Institutet, Stockholm, Sweden; c Department of Medicine, Region Västra Götaland, Sahlgrenska University Hospital, Gothenburg, Sweden; d Department of Psychology, Sensory Cognitive Interaction Laboratory (SCI-Lab), Stockholm University, Sweden; e Department of Psychology, Faculty of Health Sciences, University Fernando Pessoa Canarias, Las Palmas de Gran Canaria, Spain; f Department of Radiology, Karolinska University Hospital, The Institution for Clinical Neuroscience, Karolinska Institutet, Stockholm, Sweden; g Department of Radiology, Stanford University Hospital, Stanford, CA, USA; h Department of Neuropsychiatry, Sahlgrenska University Hospital, Region Västra Götaland, Mölndal, Sweden; i Department of Neuroimaging, Centre for Neuroimaging Sciences, Institute of Psychiatry, Psychology, and Neuroscience, King's College London, London, UK; j Department of Psychiatry, Cognition and Old Age Psychiatry, Sahlgrenska University Hospital, Region Västra Götaland, Mölndal, Sweden

**Keywords:** Body composition, Sarcopenia, Neuroimaging, Dual-energy X-ray absorptiometry, Muscle mass, Visceral adipose tissue

## Abstract

**Background:**

A better understanding of body-brain links may provide insights on targets for preventing cognitive decline. The aim was to explore associations of body composition with neuroimaging biomarkers and cognitive function among dementia-free 70-year-olds.

**Methods:**

Dual-energy X-ray absorptiometry body composition measures in relation to neuroimaging measures of cortical thickness, hippocampal volume, small vessel disease, predicted brain age, and cognitive performance were explored in a cross-sectional study of 674 dementia-free 70-year-olds from the Swedish Gothenburg H70 Birth Cohort study. Linear or ordinal regression analyses were performed.

**Findings:**

Higher quantity of muscle mass was associated with lower predicted brain age (β: −0.31 [95% CI: −0.45, −0.16], *p*: 0.00013). Those with normal level muscle mass (>7.0 men, >5.5 women kg/height m^2^) had overall thicker cortex (β: 0.043 [95% CI: 0.023, 0.064], *p*: 0.00016), thicker cortex in Alzheimer's disease signature regions (β: 0.051 [95% CI: 0.025, 0.076], *p*: 0.00040), and larger hippocampal volume (β: 111.52 [95% CI: 25.28, 197.75], *p*: 0.030) compared to those with sarcopenic level muscle mass. Higher accumulation of visceral fat was associated with overall thinner cortex (β: −0.017 [95% CI: −0.028, −0.005], *p*: 0.024). Faster gait speed and higher handgrip strength were associated with indicators of better brain health.

**Interpretation:**

Improving muscle mass fitness and lower visceral fat may be beneficial for brain health. Intervention studies are needed to confirm that targeting body composition can promote healthy brain ageing and reduce the risk of cognitive impairment among older adults.

**Funding:**

The 10.13039/501100004359Swedish Research Council, 10.13039/501100003792Hjärnfonden, and 10.13039/501100008599Alzheimerfonden.

**Research in context**

**Evidence before this study**

Traditional search sources such as PubMed were used to review the literature from inception until January 2024. We searched for studies among dementia-free older adults that investigated associations between bioimpedance, and/or dual-energy X-ray absorptiometry, and/or imaging measures of lean mass, fat mass and visceral adipose tissue, as well as components of sarcopenia (muscle mass, muscle strength and function) in relation to cognitive function, and/or predicted brain age and/or neurodegenerative and/or cerebrovascular brain magnetic resonance imaging measures. Previous research has explored the relationship between body composition and cognitive performance, but findings are inconsistent regarding links between muscle mass, body fat distribution, and markers of brain integrity. Some studies report no associations, while others linked higher visceral fat, total body fat, lower lean mass, and sarcopenia with markers of brain atrophy, such as a thinner cortex, smaller brain volume, and more white matter lesions.

**Added value of this study**

This study adds value by contributing with insights on associations between body composition measures of visceral fat, total body fat, muscle mass, strength and function in relation to brain magnetic resonance imaging markers of predicted brain age, overall cortical thickness, cortical thickness in Alzheimer's disease-related regions, hippocampal volume, and cerebral small vessel disease burden, as well as with cognitive function, among older adults free from dementia.

**Implications of all the available evidence**

The results from this study provide insights into body-brain links in ageing that may be useful for preventive strategies to promote healthy brain ageing, as factors related to body composition may be modifiable. Furthermore, insights from this study can be of value for future studies exploring the impact of body composition on cognitive decline and dementia risk among older adults.

## Introduction

Alterations in body composition, brain structures, and cognitive function occur with ageing.^1^^,^^2^ Body fat increases and muscle mass decreases with ageing, thus increasing the risk of age-related conditions such as sarcopenia, characterised by the loss of muscle mass, function, and strength.^2^ To some extent, decline in certain brain areas and cognitive function can be expected as people age, but it can also signal pathological changes related to accumulating neurodegenerative diseases such as Alzheimer's disease (AD) and related disorders.^3^

Among older adults (>65 years), low muscle mass, sarcopenia, and co-occurrence of low muscle mass and excess adiposity (sarcopenic obesity) have been associated with cognitive decline and dementia risk.^4^^,^^5^ It has been proposed that factors (e.g., inflammation, metabolic alterations, and neurological factors) related to muscle mass, strength and function may be involved,^6^^,^^7^ and that excess body fat and visceral fat accumulation may increase the risk of cognitive decline and dementia by contributing to brain lesions through vascular and metabolic pathways.^8^ It has also been suggested that low muscle mass combined with excess body fat may have a synergistic effect, as this combination has been associated with smaller brain volumes compared to either condition alone.^9^

Muscle strength and function have been associated with brain structures and cognitive function.^10^^,^^11^ However, to what extent age-related changes in body composition are associated with brain and cognitive health is poorly understood. Exploring the impact of body fat distribution and muscle mass in relation to neuroimaging biomarkers (e.g., cortical thickness, hippocampal volume, predicted brain age, and markers of small vessel disease) could provide insights on body-brain links that may impact the risk of cognitive disorders. These insights can be valuable for preventive strategies to promote healthy brain ageing, as factors related to body composition may be modifiable. Yet, limited research exists on these associations, yielding mixed results. Previous studies have found either no associations, or that higher muscle quantity was associated with larger whole brain, grey matter, and white matter volumes,^12^^,^^13^ while higher body fat percentage and greater accumulation of visceral adipose tissue (VAT) were associated with indicators of cerebral small vessel disease (CSVD),^13^^,^^14^ smaller grey matter volume,^15^ thinner cortex,^16^ and higher (older) predicted brain age.^17^^,^^18^

The primary aim of this cross-sectional study was to explore the hypothesis that lower muscle mass, higher total body fat percentage, and higher accumulation of VAT are related to indicators of lower brain integrity and cognitive function among dementia-free older adults. To assess muscle quantity and body fat distribution, we used Dual X-ray absorptiometry (DXA) measures of lean mass, total body fat percentage, and VAT. Structural brain magnetic resonance imaging (MRI) measures of cortical thickness (global, and AD-signature cortical regions), hippocampal volume, CSVD burden, and predicted brain age were included as indicators of brain integrity. Cognitive performance was assessed with a global cognitive composite score covering five cognitive domains. A secondary aim was to investigate associations of muscle strength and function (measured with hand grip strength and gait speed) with the brain MRI measures and cognitive performance.

## Methods

**Fig. 1:**
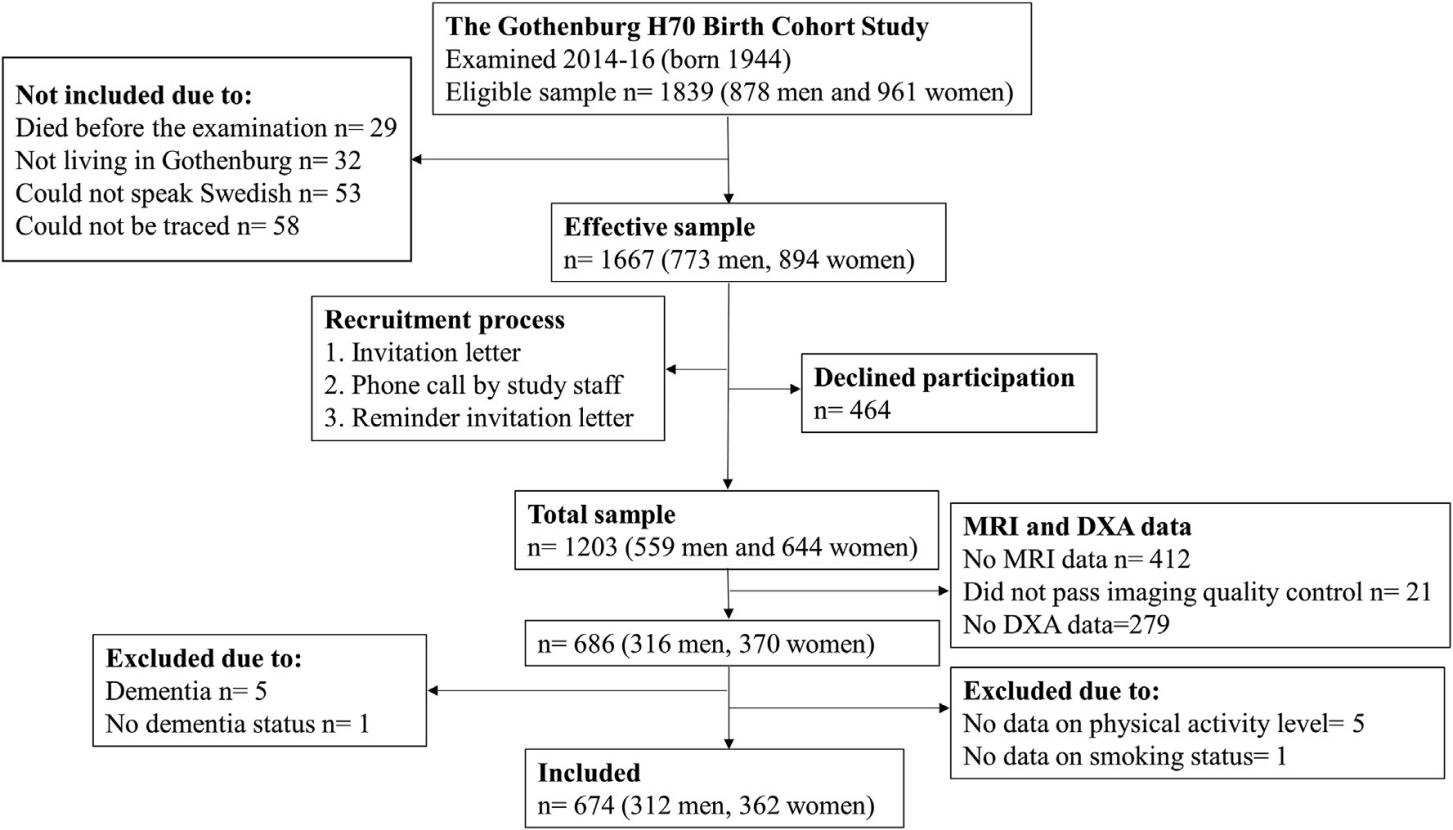
Participant flowchart.

Cross-sectional data were derived from the Swedish population-based Gothenburg H70 Birth Cohort Study (the H70-study), described in detail previously.^19^ In order to include a representative sample, all 70-year-olds living in Gothenburg and born 1944 on birth dates ending with 0, 2, 5 or 8 were invited to participate (n = 1839). Data were collected in 2014–16 on the 1203 participants that accepted participation. The study comprised a one-day general examination at the Neuropsychiatric Clinic at Sahlgrenska University Hospital, as well as a number of additional examinations. A clinical cognitive examination was performed as part of the general examination on 1196 participants. All participants were invited to participate in the additional MRI and DXA examinations. There were 791 individuals that performed an MRI examination. Non-participation was due to several reasons (e.g., declining participation, no response or expressing fear or discomfort). For those willing to undergo MRI, five could not participate due to contraindications (i.e. pacemakers). The examination was interrupted on 22 occasions due to claustrophobia and three occasions due to noise discomfort. The DXA examination was performed in 993 individuals. Non-participants (n = 210) did not perform the examination due to several reasons (e.g., declining participation, or technical reasons or no contact). We excluded participants with dementia from the DXA and MRI sample (n = 5). The final sample comprised of 674 dementia-free participants with MRI and DXA data. See participant flowchart in Fig. 1.

### Ethics

The study was approved by the Regional Ethics Review Board in Gothenburg, Sweden (Reference number 869-13). The participants gave written informed consent to participate in the study, and for their data to be used for research purposes, according to the Helsinki declaration.

### DXA acquisition and measurement

Body composition was measured by Dual-energy X-ray absorptiometry (DXA) using a Lunar Prodigy scanner. Whole-body scans were performed, and lean soft tissue and fat mass was analysed (enCORE software version 18.30.006).

Appendicular lean soft tissue index (ALSTI), defined as the sum of lean soft tissue in arms and legs divided by body height in meters squared (m^2^), was used as an estimate of muscle mass.

Fat mass was measured as total body fat percentage, and VAT was measured in kg.

### Markers of physical function

Handgrip strength was measured with a Martin Vigorimeter (Gebrüder Martin GmbH & Co, Tuttlingen, Germany).^19^ The large bulb was used for men and the medium bulb for women. The test was repeated three times for each hand, and the highest value of the strongest hand was used (measured in kPa). Self-selected gait speed was measured over 30-m indoors with standing start (meter/second).

### Sarcopenia

Probable sarcopenia was estimated by using cutoffs for handgrip strength, and sarcopenia was estimated by using cutoffs for handgrip strength and muscle mass. Cutoffs to classify sarcopenic level muscle mass were: ALSTI <7.0 for men and <5.5 for women (kg/body height m^2^).^2^ Those with values above the ALSTI cutoffs were labelled having “normal” level muscle mass. Cutoffs used to classify probable sarcopenia were handgrip strength <59 kPa for women, and <69 kPa for men.^20^ Severe sarcopenia was not appraised as a previous study reported that there were no participants with severe sarcopenia in this study sample (estimated by low muscle mass, low muscle strength and low physical performance).^20^

### Brain MRI acquisition

All participants were scanned on a 3.0T Philips Achieva system (Philips Medical Systems). The imaging protocol included a three-dimensional (3D) T1-weighted Turbo Field Echo (TFE) sequence to assess structural changes, a T2-weighted sequence to exclude other pathologies (e.g., tumours) and to evaluate enlarged perivascular spaces, a fluid-attenuated inversion recovery (FLAIR) sequence for detecting white matter hyperintensities (WMH) and lacunes, and venous bold sequence (VenoBOLD) for the detection of microbleeds.^19^ Cortical reconstruction and volumetric segmentation of subcortical grey matter volumes were performed on the T1-weighted images using the FreeSurfer 7.2 image analysis pipeline.

Small vessel disease markers were defined according to the Standards for Reporting Vascular Changes.^21^ Lacunes (3–15 mm), enlarged perivascular spaces (PVS), and cerebral microbleeds (CMBs) were assessed by an experienced radiologist blinded to clinical data. WMH volumes were automatically segmented with the Lesion Segmentation Tool 2.0.15 using a lesion prediction algorithm running under the statistical parametric mapping software, SPM12.

Biological brain age is a theoretical concept of a measurement developed to track the state of a person's biophysiological brain ageing, in an attempt to unveil why individuals with the same chronological age present different atrophy patterns. The brain age model is based on a convolutional neural network, trained on 18,843 raw T1-weighted brain MRIs of cognitively unimpaired individuals, with an average age of 65.4 ± 9.0 years, from 7 different cohorts (AddNeuroMed, ADNI, AIBL, Genic, UK Biobank and the H70-study), using a 10-fold cross-validation approach. Images were only rigid registered to the Montreal Neuroscience Institute (MNI) atlas space before being used as input to the model. Four approaches were used to validate the brain age model: 1) the hold-out method, 2) advanced 10-fold cross-validation with two external cohorts (AddNeuroMed and J-ADNI), 3) adding external data during training in the cross-validation, and 4) cross-validation with skull-stripped brain images. The third approach resulted in the smallest Mean Absolute Error (MAE), which was therefore used in the current manuscript. The models showed MAE levels comparable to previous studies, indicating strong performance with the advantage of requiring only one preprocessing step, described in detail previously.^22^ No age bias correction was necessary as all participants were 70 years old.

### Brain MRI markers

Total mean cortical thickness was derived by averaging cortical thickness of the right and left hemisphere. An AD-signature cortical thickness measure was derived by averaging cortical thicknesses of entorhinal, inferior temporal, middle temporal, and fusiform regions (AD-signature meta-regions of interest, ROI) from the Desikan-Killiany Atlas.^23^, ^24^, ^25^ The AD-signature regions were adjusted for surface area by (where *i* represents the index of the regions of interest):

$$\frac{\sum_{i=1}^{i=4}\;\left(\frac{{cortical\;area}_{i}}{\sum_{i=1}^{i=4}{cortical\;area}_{i}}\;\times \;100\right)\;\times \;{cortical\;thickness}_{i}}{100}$$

This adjustment was performed to account for that the regions included in the AD-signature meta-ROI don't have the same size.^23^

Mean hippocampal volume was adjusted for total intracranial volume (TIV). Adjustment was performed by using residuals of a least-square derived linear regression between hippocampal volume (*y*) and TIV (*x*).

The brain age difference is calculated individually, also known as the “brain age gap” (BAG), which is the difference between the individual's chronological age and the predicted brain age. A negative value indicates a biologically younger brain, and a positive value a biological older brain, compared to chronological age.

A CSVD score including measures of WMH volume, CMBs, enlarged PVS in basal ganglia, and lacunes was generated as a measure of overall cerebrovascular burden.^26^ WMH volume was adjusted for TIV by using residuals of a least-square derived linear regression between WMH volume (y) and TIV (x). WMH were categorised by computing quartiles. One point was awarded if WMH were present in the third or fourth quartiles. Presence of ≥1 lacune or ≥1 CMB was each awarded with one point. PVS in the basal ganglia were awarded with one point for ≥11 PVS. The CSVD score was constructed by summing WMH, CMB, PVS, and lacunes as an ordinal score of 0–4. To account for low power, SVD scores 3 and 4 were combined for the analysis (n = 35). The final CSVD score ranged between 0 (low burden) to 3 (high burden).

### Dementia diagnosis

Dementia diagnosed according to the Diagnostic and statistical manual of mental disorders (3rd ed., Revised (DSM–III–R)), was an exclusion criterium.^19^^,^^27^

### Assessment of cognitive function

The cognitive examination was a semi-structured interview conducted by a psychiatric nurse, a psychiatrist, or a medical doctor.^19^ Cognitive performance was measured using a global cognitive composite score,^28^ derived from eight cognitive tests covering five cognitive domains: executive function (Digit Span Backward and Figure Logic), perceptual speed (Figure Identification-Psif), verbal fluency (phonemic fluency derived from the Controlled Oral Word Association-FAS and semantic fluency derived from “animals” task), episodic memory (12-object recall, and 12-object delayed recall), and visuospatial abilities (Koh's block test).^19^^,^^29^ The participants had to have information on at least three cognitive tests to be included. The global cognitive composite score was constructed by dividing the summarised z-scores by number of tests. Cognitive function was also rated in accordance with a Swedish version of the Mini-Mental State Examination (MMSE).

### Characteristics and potential confounders

Information on characteristics and potential confounders were obtained through semi-structured interviews and health examinations, described in detail previously.^19^ Potential confounders were chosen based on theory and identified with a causal directed acyclic graph (DAG) (Supplementary Figure S1). Biological sex, educational level, physical activity level, and smoking were considered potential confounders. Age was not added as a confounder as all participants were examined at age 70 years. Sex was identified by the Swedish personal identity number. A Swedish personal identity number is a unique identification number allocated to a person when they are born, or when they move to Sweden. Educational level was divided into three categories based on Swedish education classifications: compulsory primary education, secondary education, and higher education. Smoking was defined as either smoking or not smoking. Physical activity was divided into four categories based on an updated version of the Saltin-Grimby physical activity level scale; physically inactive, some light physical activity, regular physical activity and training, and regular hard physical training.^30^ Physical activity was dichotomised into inactive/light physical activity or regular physical activity/hard physical training.

### Statistics

To examine if the sample size of our data set was sufficient to detect medium-sized relationships, the Green's formula for linear regression was applied, i.e. N > 50 + 8∗m, where N is the minimal required sample size and m the number of independent variables.^31^^,^^32^ With a maximum of eight independent variables in the statistical models, we needed N > 114 observations, which was fulfilled given the sample size of 674 in this study.

The student's independent t-test (normally distributed continuous variables: age, brain age, cortical thickness measures, body fat percentage, hippocampal volume, gait speed, handgrip strength, BMI, and ALSTI), the Mann–Whitney U test (MMSE score, global cognitive composite score, BAG, and VAT), and the Chi-square test (categorical variables: CSVD score, education, sarcopenia, probable sarcopenia, ALSTI dichotomised, physical activity level, and smoking) were performed to compare characteristics between men and women. Continuous variables were presented as means (SD) or medians (IQR), and proportions as number and percent (%).

Analyses were performed with linear or ordinal logistic regression models to explore cross-sectional associations of body composition measures with brain MRI measures and cognitive performance. Dependent variables in the linear regression models were total mean cortical thickness, mean cortical thickness in AD-signature regions, hippocampal volume, predicted brain age (estimated as BAG), and a global cognitive composite score. Dependent variable in the ordinal logistic regression model (cumulative logit model) was the CSVD score. Independent variables were markers of muscle mass (ALSTI, and ALSTI dichotomised), total body fat percentage, VAT (kg), handgrip strength (kPa), gait speed (meter/second), and probable sarcopenia (yes/no). In the regression analyses, ALSTI (continuous) was adjusted for sex by using the residuals of a linear regression with appendicular lean soft tissue in kg as the outcome variable, and interaction variables of sex and body height as predictors. The body composition measures ALSTI (sex-adjusted, continuous), body fat percentage, and VAT were analysed in the same model, with results reported separately for each body composition measure. Probable sarcopenia, handgrip strength, and gait speed were investigated separately in relation to the MRI measures and the global cognitive composite score. All analyses were performed in two models, where model 1 was adjusted for sex, and model 2 was adjusted for sex, education, smoking, and physical activity level. The analyses investigating ALSTI dichotomised (sarcopenic level muscle mass/normal level muscle mass) in relation to the MRI measures and cognitive performance were adjusted for sex, body fat percentage, and VAT in model 1, and for sex, body fat percentage, VAT, education, smoking, and physical activity level, in model 2. Sarcopenia (estimated by low muscle mass and low muscle strength) was not investigated due to low prevalence (n = 16, 2.3%).

Assumptions underlying the linear regression models were controlled. Normality was assessed by examining p–p plots and histograms of the residuals. Multicollinearity was tested by Variance Inflation Factor (VIF was <4) and by examining the correlation matrix of predictors. The linearity assumption was tested with scatterplots of the linear relationship between the quantitative independent variables and dependent variables. For the ordinal logistic regression model, the proportional odds assumptions were controlled with a likelihood ratio test, and linearity for quantitative predictors were assessed by creating scatterplots of each quantitative predictor against the ordinal response variable.

Differences in body composition between men and women were considered when constructing the body composition variables (e.g., different cutoffs for sarcopenic level muscle mass and probable sarcopenia), and by adjusting the regression models for sex. Furthermore, potential interactions between biological sex and ALSTI, VAT, and body fat percentage (interaction variables: sex∗body composition measure + sex + body composition measure) were explored in relation to the brain MRI markers and the global cognitive composite score with linear or ordinal regression models adjusted for ALSTI, VAT, body fat percentage and potential confounders. If an interaction (*p* < 0.05) was detected, results were presented by sex. Results from the analyses performed stratified by sex were reported in Supplementary Material.

Results from the regression models were interpreted based on estimates (β-Coefficients for the linear regression models, and OR for the ordinal logistic regression model) with 95% CI. False discovery rate (FDR) correction was applied to explore potential Type 1 errors in models 2 of the main linear and ordinal regression analyses.

The statistical analyses were performed in IBM SPSS STATISTICS 29, and R programming version 4.1.2.

### Role of funders

The funding sources had no role in the study design, data collection, data analyses, interpretation, writing of the manuscript or the decision to submit it for publication. The authors have not been paid to write this article by a pharmaceutical company or other agency.

## Results

Sample characteristics are presented in Table 1. There were 362 (54%) women, and 312 (46%) men included in this study. Women had higher mean body fat percentage (40.5% vs. 31.4%), lower mean VAT (0.8 kg vs. 1.7 kg), less often muscle mass at a sarcopenic level (6% vs. 13%), and lower handgrip strength (73.2 kPa vs. 84.8 kPa) compared to men. There was no evidence of a difference in mean gait speed (1.31 m/s vs. 1.32 m/s) between women and men. The prevalence of sarcopenia (estimated by low muscle mass and low muscle strength) was low among both women (1.2%) and men (3.5%), and there was no evident difference in proportion women and men with probable sarcopenia (14%).

**Table 1:** Sample characteristics of dementia-free participants from the Gothenburg H70 Birth Cohort Study.

	Total sample N = 674	Women N = 362	Men N = 312	*p-*value
	Mean (SD)	Mean (SD)	Mean (SD)	
**Age (**years)	70.8 (0.3)	70.8 (0.3)	70.8 (0.3)	0.88
**Predicted brain age (**years)	71.5 (1.8)	71.4 (1.7)	71.5 (1.9)	0.99
**Total body fat (**%)	36.3 (8.0)	40.5 (6.9)	31.4 (6.2)	<0.0001
**Total mean cortical thickness (**mm)	2.35 (0.08)	2.35 (0.07)	2.35 (0.08)	0.032
**AD-signature cortical thickness**^b^ (mm)	2.72 (0.09)	2.72 (0.09)	2.73 (0.10)	0.52
**Hippocampal volume** (adjusted for total intracranial volume, mm^3^)	3931 (320)	3923 (290)	3941 (351)	0.47
**Handgrip strength (**kPa)	78.6 (16.1)	73.2 (14.0)	84.8 (16.2)	<0.0001
**Gait speed (**meter/second)	1.31 (0.17)	1.31 (0.17)	1.32 (0.17)	0.27
**BMI (**kg/m^2^)	26.2 (4.3)	26.1 (4.8)	26.3 (3.7)	0.66
**ALSTI**^c^	7.17 (1.04)	6.56 (0.78)	7.87 (0.84)	<0.0001

	**Median (IQR)**	**Median (IQR)**	**Median (IQR)**	
**MMSE score (**0–30)	29 (29, 30)	30 (29, 30)	29 (29, 30)	0.068
**Global cognitive composite score**^c^ (z-score)	0.13 (−0.30, 0.54)	0.20 (−0.20, 0.57)	0.04 (−0.40, 0.51)	0.0039
**Visceral adipose tissue (**kg)	1.2 (0.6, 1.9)	0.8 (0.4, 1.3)	1.7 (1.1, 2.2)	<0.0001
**Brain age gap**^a^ (years)	0.30 (−0.60, 1.40)	0.45 (−0.60, 1.50)	0.30 (−0.50, 1.4)	0.55

	**N (%)**	**N (%)**	**N (%)**	
**Sarcopenic level muscle mass**^d^ (ALSTI <7.0 men, <5.5 women)	61 (9)	21 (6)	40 (13)	0.0016
**Probable sarcopenia** (Handgrip strength <69 men, <59 women)	94 (14)	51 (14)	43 (14)	0.90
**Sarcopenia** (low muscle mass and low handgrip strength)	16 (2.3)	5 (1.2)	11 (3.5)	0.069
**Cerebral small vessel disease score** (0–3)^e^				0.0026
0	265 (40)	125 (35)	140 (45)	
1	263 (39)	164 (46)	99 (32)	
2	107 (16)	56 (16)	51 (17)	
3	35 (5)	15 (4)	20 (7)	
**Physical activity** (Regular physical activity)	589 (87)	315 (87)	274 (88)	0.75
**Education**				<0.0001
Compulsory primary education	52 (8)	21 (6)	31 (10)	
Secondary education	293 (44)	188 (52)	105 (34)	
Higher education	329 (49)	153 (42)	176 (56)	
**Smoking**^c^ (Non-smoking)	632 (94)	335 (93)	297 (95)	0.16

Note: Educational level was divided into three categories based on Swedish education classifications: compulsory primary education (pre-primary education, primary and lower secondary education less than 9 years), secondary education (primary and lower secondary education 9 years, upper secondary education, and post-secondary education less than 2 years), and higher education (post-secondary education 2 years or longer, and postgraduate education). Smoking was defined as either smoking (current smoker) or non-smoking (previous smoker or never smoked). Physical activity was dichotomized into inactive/light physical activity or regular physical activity. BMI was measured as kg body weight/body height meter^2^. There were missing cerebral small vessel disease (CSVD) data for 4 participants, missing MMSE (Mini-Mental State Examination) data for 2 participants, missing gait speed data for 10 participants, missing visceral adipose tissue (VAT) data for 3 participants, and missing hand grip strength data for 1 participant.

a Brain age gap (BAG) is the difference between chronological age and predicted brain age. A negative value indicates a biologically younger brain, and a positive value a biological older brain, compared to chronological age.

b AD-signature: Averaging cortical thicknesses of entorhinal, inferior temporal, middle temporal, and fusiform regions, adjusted for surface area.

c The global cognitive composite score was derived from eight cognitive tests covering five cognitive domains: executive function, perceptual speed, verbal fluency, episodic memory, and visuospatial abilities.

d Appendicular lean soft tissue index (ALSTI, kg/body height m^2^) is used as a marker of muscle mass. Sarcopenic level muscle mass: below the EWGSOP2 sarcopenia cut-off of ALSTI <7.0 men, <5.5 women.

e The global cerebral small vessel disease score ranged from 0 (no cerebrovascular burden)—3 (high cerebrovascular burden), including measures of white matter hyperintensities, cerebral microbleeds, enlarged perivascular spaces in basal ganglia, and lacunes.

### Linear regression β-Coefficients with 95% CI for associations of body composition measures, handgrip strength, and gait speed with cortical thickness

Compared to participants with sarcopenic level muscle mass, those with normal level muscle mass had overall thicker cortex (Model 2, β: 0.043 [95% CI: 0.023, 0.064], *p*: <0.0001, FDR corrected *p*: 0.00016), and thicker cortex in AD-signature regions (Model 2, β: 0.051 [95% CI: 0.025, 0.076], *p*: 0.00010, FDR corrected *p*: 0.00040). No evidence associating muscle mass with total mean cortical thickness, or cortical thickness in AD-signature regions, were detected when muscle mass (ALSTI) was investigated as a continuous variable (Table 2). Higher accumulation of VAT was associated with lower total mean cortical thickness (Model 2, β: −0.017 [95% CI: −0.028, −0.005], *p*: 0.0061, FDR corrected *p*: 0.024). There was an association between higher accumulation of VAT and thinner cortex in AD-signature regions, but the association did not remain after adjusting for multiple comparisons (Model 2, β: −0.018 [95% CI: −0.032, −0.003], *p*: 0.017, FDR corrected *p*: 0.069) (Table 2). There was an association between higher body fat percentage and overall thicker cortex (Model 2, β: 0.002 [95% CI: 0.000, 0.003], *p*: 0.036, FDR corrected *p*: 0.096). However, this association did not remain after adjusting for multiple comparisons, and there was no evidence of an association in model 1. Nor was there evidence of an association between total body fat percentage and cortical thickness in AD-signature regions (Table 2). Furthermore, there was an association between faster gait speed and overall thicker cortex that did not remain after adjusting for multiple comparisons (Model 2, β: 0.038 [95% CI: 0.001, 0.075], *p*: 0.047, FDR corrected *p*: 0.094) (Table 3). There was no evidence of associations between either hand grip strength or probable sarcopenia in relation to the cortical thickness measures (Tables 3 and 4).

**Table 2:** Linear regression β-Coefficients or ordinal logistic regression OR with 95% confidence intervals for associations of body composition with neuroimaging biomarkers and cognitive function.

N = 674	Model 1	*p*-value	Model 2	*p*-value	*p*-value adjusted^a^
	β (95% CI)		β (95% CI)		
**Total mean cortical thickness**^c^					
Appendicular lean soft tissue index (ALSTI)^b^	0.003 (−0.003, 0.010)	0.32	0.003 (−0.003, 0.010)	0.31	0.35
Normal level muscle mass compared to sarcopenic level muscle mass^b^	0.043 (0.023, 0.063)	<0.0001	0.043 (0.023, 0.064)	<0.0001	0.00016
Total body fat percentage	0.001 (0.000, 0.003)	0.071	0.002 (0.000, 0.003)	0.036	0.096
Visceral adipose tissue	−0.017 (−0.029, −0.005)	0.0045	−0.017 (−0.028, −0.005)	0.0061	0.024
**AD-signature cortical thickness**^c^					
Appendicular lean soft tissue index (ALSTI)^b^	0.004 (−0.004, 0.012)	0.34	0.004 (−0.004, 0.012)	0.29	0.39
Normal level muscle mass compared to sarcopenic level muscle mass^b^	0.048 (0.023, 0.073)	0.00017	0.051 (0.025, 0.076)	0.00010	0.00040
Total body fat percentage	0.001 (−0.001, 0.003)	0.22	0.001 (0.000, 0.003)	0.15	0.25
Visceral adipose tissue	−0.018 (−0.032, −0.003)	0.017	−0.018 (−0.032, −0.003)	0.017	0.069
**Hippocampal volume**^c^					
Appendicular lean soft tissue index (ALSTI)^b^	24.33 (−1.99, 50.66)	0.070	22.08 (−4.27, 48.43)	0.10	0.16
Normal level muscle mass compared to sarcopenic level muscle mass^b^	126.39 (40.90, 211.89)	0.0038	111.52 (25.28, 197.75)	0.011	0.030
Total body fat percentage	5.00 (−0.85, 10.85)	0.094	6.21 (0.31, 12.11)	0.039	0.10
Visceral adipose tissue	−32.28 (−81.25, 16.69)	0.20	−25.77 (−74.64, 23.10)	0.30	0.40
**Predicted brain age**^e^					
Appendicular lean soft tissue index (ALSTI)^b^	−0.33 (−0.47, −0.18)	<0.0001	−0.31 (−0.45, −0.16)	<0.0001	0.00013
Normal level muscle mass compared to sarcopenic level muscle mass^b^	−0.84 (−1.32, −0.37)	0.00050	−0.72 (−1.20, −0.24)	0.0031	0.0082
Total body fat percentage	−0.026 (−0.058, 0.006)	0.12	−0.035 (−0.067, −0.002)	0.036	0.058
Visceral adipose tissue	0.347 (0.077, 0.617)	0.012	0.299 (0.031, 0.567)	0.029	0.057
**Global cognitive composite score**^d^					
Appendicular lean soft tissue index (ALSTI)^b^	0.028 (−0.022, 0.077)	0.27	0.013 (−0.033, 0.060)	0.58	0.58
Normal level muscle mass compared to sarcopenic level muscle mass^b^	0.101 (−0.060, 0.262)	0.22	0.024 (−0.130, 0.178)	0.76	0.76
Total body fat percentage	−0.010 (−0.021, 0.001)	0.078	−0.006 (−0.017, 0.004)	0.25	0.37
Visceral adipose tissue	−0.066 (−0.158, 0.026)	0.16	−0.038 (−0.125, 0.049)	0.39	0.45

	**OR (95% CI)**	***p*-value**	**OR (95% CI)**	***p*-value**	***p*-value adjusted**^a^
**Cerebral small vessel disease score**^f^					
Appendicular lean soft tissue index (ALSTI)^b^	0.86 (0.74, 1.01)	0.061	0.89 (0.76, 1.04)	0.13	0.16
Normal level muscle mass compared to sarcopenic level muscle mass^b^	0.51 (0.31, 0.83)	0.0070	0.58 (0.35, 0.97)	0.036	0.064
Total body fat percentage	1.00 (0.96, 1.03)	0.83	0.99 (0.96, 1.03)	0.61	0.61
Visceral adipose tissue	1.46 (1.10, 1.94)	0.010	1.41 (1.06, 1.88)	0.019	0.071

Note: Analyses were performed with linear or ordinal logistic regression models. Dependent variables in the linear regression models were total mean cortical thickness, mean cortical thickness in AD-signature regions, hippocampal volume, predicted brain age, and a global cognitive composite score. Dependent variable in the ordinal logistic regression model (cumulative logit model) was the cerebral small vessel disease (CSVD) score. The independent variables ALSTI, total body fat percentage and Visceral adipose tissue (VAT) were investigated in the same model, with results reported separately for each body composition measure. Model 1 was adjusted for sex. Model 2 was adjusted for sex, physical activity, education, and smoking. ALSTI dichotomised (normal level muscle mass/sarcopenic level muscle mass) was investigated in a separate model where model 1 was adjusted for sex, VAT, and body fat percentage, and model 2 was adjusted for sex, VAT, body fat percentage, physical activity, education, and smoking. There was missing VAT data for 3 participants (0.4%), and missing CSVD data for 4 participants (0.5%).

a FDR (false discovery rate) adjusted *p*-values were added to explore the impact of conducting multiple comparisons (including the total number of analyses reported in Table 2, Table 3, Table 4) for model 2.

b ALSTI: Appendicular lean soft tissue index (kg/body height m^2^) was used as a marker of muscle mass. In the analyses, ALSTI (continuous) was adjusted for sex by using the residuals of a linear regression model with appendicular lean soft tissue in kg as the outcome variable, and interaction variables of sex and body height as predictors. Sarcopenic level muscle mass was classified as: below the EWGSOP2 sarcopenia cut-off of ALSTI <7.0 for men, and <5.5 for women, and normal level muscle mass was classified as levels above the ALSTI cut-offs.

c Total mean cortical thickness is measured in mm. Alzheimer's disease (AD)-signature: Averaging cortical thicknesses of entorhinal, inferior temporal, middle temporal, and fusiform regions, adjusted for surface area, measured in mm. Hippocampal volume was adjusted for total intracranial volume, measured in mm^3^.

d The global cognitive composite score (z-score) was derived from eight cognitive tests covering five cognitive domains: executive function, perceptual speed, verbal fluency, episodic memory, and visuospatial abilities.

e Predicted brain age was investigated by the difference in years between chronological age and predicted biological age (brain age gap, BAG). A negative value indicates a biologically younger brain, and a positive value a biological older brain, compared to chronological age.

f The CSVD score ranged from 0 (no cerebrovascular burden)—3 (high cerebrovascular burden).

**Table 3:** Linear regression β-Coefficients or ordinal logistic regression OR with 95% confidence intervals for associations of hand grip strength and gait speed with neuroimaging biomarkers and cognitive function.

N = 674	Model 1	*p*-value	Model 2	*p*-value	*p*-value adjusted^a^
	β (95% CI)		β (95% CI)		
**Total mean cortical thickness**^d^					
Hand grip strength^b^	0.0002 (−0.0002, 0.0006)	0.30	0.0002 (−0.0002, 0.0006)	0.37	0.37
Gait speed^c^	0.039 (0.005, 0.074)	0.026	0.038 (0.001, 0.075)	0.047	0.094
**AD-signature cortical thickness**^d^					
Hand grip strength^b^	0.0003 (−0.0002, 0.0007)	0.28	0.0003 (−0.0002, 0.0007)	0.29	0.47
Gait speed^c^	0.042 (0.000, 0.084)	0.052	0.046 (0.000, 0.092)	0.052	0.16
**Hippocampal volume**^d^					
Hand grip strength^b^	2.25 (0.65, 3.85)	0.0060	2.00 (0.39, 3.61)	0.015	0.044
Gait speed^c^	214.59 (71.10, 358.08)	0.0034	174.61 (18.96, 330.27)	0.028	0.084
**Predicted brain age**^f^					
Hand grip strength^b^	−0.010 (−0.019, −0.001)	0.022	−0.008 (−0.017, 0.001)	0.073	0.15
Gait speed^c^	−1.11 (−1.91, −0.31)	0.0070	−0.52 (−1.38, 0.35)	0.24	0.36
**Global cognitive composite score**^e^					
Hand grip strength^b^	0.003 (0.000, 0.006)	0.085	0.001 (−0.002, 0.004)	0.36	0.43
Gait speed^c^	0.595 (0.327, 0.863)	<0.0001	0.341 (0.065, 0.618)	0.016	0.019

	**OR (95% CI)**	***p*-value**	**OR (95% CI)**	***p*-value**	***p*-value adjusted**^a^
**Cerebral small vessel disease score**^g^					
Hand grip strength^b^	0.99 (0.98, 1.00)	0.12	1.00 (0.99, 1.01)	0.28	0.36
Gait speed^c^	0.20 (0.09, 0.47)	0.00020	0.31 (0.12, 0.77)	0.011	0.043

Note: Analyses were performed with linear or ordinal logistic regression models. Dependent variables in the linear regression models were total mean cortical thickness, mean cortical thickness in AD-signature regions, hippocampal volume, predicted brain age, and a global cognitive composite score. Dependent variable in the ordinal logistic regression model (cumulative logit model) was the cerebral small vessel disease (CSVD) score. Independent variables were hand grip strength and gait speed. Hand grip strength and gait speed were investigated in separate analyses. Model 1 was adjusted for sex, and model 2 was adjusted for sex, physical activity, education, and smoking. There were missing CSVD data for 4 participants, missing gait speed data for 10 participants (1.4%), and missing hand grip strength data for 1 participant (0.1%).

a FDR (false discovery rate) adjusted *p*-values were added to explore the impact of conducting multiple comparisons (including the total number of analyses reported in Table 2, Table 3, Table 4) in model 2.

b Handgrip strength (kPa), best value out of three on the dominant hand.

c Gait speed self-selected 30 m indoor (meter/second).

d Total mean cortical thickness is measured in mm. Alzheimer's disease (AD)-signature: Averaging cortical thicknesses of entorhinal, inferior temporal, middle temporal, and fusiform regions, adjusted for surface area, measured in mm. Hippocampal volume was adjusted for total intracranial volume, measured in mm^3^.

e The global cognitive composite score (z-score) was derived from eight cognitive tests covering five cognitive domains: executive function, perceptual speed, verbal fluency, episodic memory, and visuospatial abilities.

f Predicted brain age is investigated by the difference in years between chronological age and predicted biological age (brain age gap). A negative value indicates a biologically younger brain, and a positive value a biological older brain, compared to chronological age.

g The CSVD score ranged from 0 (no cerebrovascular burden)—3 (high cerebrovascular burden).

**Table 4:** Linear regression β-Coefficients or ordinal logistic regression OR with 95% confidence intervals for associations of probable sarcopenia with neuroimaging biomarkers and cognitive function.

N = 674	Model 1	*p*-value	Model 2	*p*-value	*p*-value adjusted^a^
	β (95% CI)		β (95% CI)		
**Total mean cortical thickness**^c^					
No probable sarcopenia compared to probable sarcopenia^b^	0.002 (−0.014, 0.019)	0.27	0.001 (−0.016, 0.018)	0.88	0.88
**AD-signature cortical thickness**^c^					
No probable sarcopenia compared to probable sarcopenia^b^	−0.002 (−0.023, 0.018)	0.83	−0.002 (−0.023, 0.019)	0.83	0.83
**Hippocampal volume**^c^					
No probable sarcopenia compared to probable sarcopenia^b^	38.71 (−30.12, 109.54)	0.27	25.94 (−44.46, 96.34)	0.47	0.65
**Predicted brain age**^e^					
No probable sarcopenia compared to probable sarcopenia^b^	−0.597 (−0.985, −0.209)	0.0030	−0.481 (−0.869, −0.093)	0.015	0.030
**Global cognitive composite score**^d^					
No probable sarcopenia compared to probable sarcopenia^b^	0.233 (0.102, 0.364)	0.00050	0.181 (0.056, 0.305)	0.0045	0.0053

	**OR (95% CI)**	***p*-value**	**OR (95% CI)**	***p*-value**	***p*-value adjusted**^a^
**Cerebral small vessel disease score**^f^					
No probable sarcopenia compared to probable sarcopenia^b^	0.075 (0.50, 1.13)	0.17	0.086 (0.57, 1.29)	0.40	0.45

Note: Analyses were performed with linear or ordinal logistic regression models. Dependent variables in the linear regression models were total mean cortical thickness, mean cortical thickness in AD-signature regions, hippocampal volume, predicted brain age, and a global cognitive composite score. Dependent variable in the ordinal logistic regression model (cumulative logit model) was the cerebral small vessel disease (CSVD) score. Independent variable was probable sarcopenia (yes/no). Model 1 was adjusted for sex, and model 2 was adjusted for sex, physical activity, education, and smoking. There were missing CSVD data for 4 participants, and missing hand grip strength data for 1 participant (0.1%).

a FDR (false discovery rate) adjusted *p*-values were added to explore the impact of conducting multiple comparisons (including the total number of analyses reported in Table 2, Table 3, Table 4) in model 2.

b Handgrip strength (kPa), best value out of three on the dominant hand. Probable sarcopenia was categorised based on handgrip strength below cutoff: <59 kPa for women and <69 kPa for men.

c Total mean cortical thickness is measured in mm. Alzheimer's disease (AD)-signature: Averaging cortical thicknesses of entorhinal, inferior temporal, middle temporal, and fusiform regions, adjusted for surface area, measured in mm. Hippocampal volume was adjusted for total intracranial volume, measured in mm^3^.

d The global cognitive composite score (z-score) was derived from eight cognitive tests covering five cognitive domains: executive function, perceptual speed, verbal fluency, episodic memory, and visuospatial abilities.

e Predicted brain age is investigated by the difference in years between chronological age and predicted biological age (brain age gap). A negative value indicates a biologically younger brain, and a positive value a biological older brain, compared to chronological age.

f The CSVD score ranged from 0 (no cerebrovascular burden)—3 (high cerebrovascular burden).

### Linear regression β-Coefficients with 95% CI for associations of body composition measures, handgrip strength, and gait speed with hippocampal volume

Participants with normal level muscle mass had larger hippocampal volume compared to those with sarcopenic level muscle mass (Model 2, β: 111.52 [95% CI: 25.28, 197.75], *p*: 0.011, FDR corrected *p*: 0.030). No evidence of associations between either ALSTI (continuous) or VAT was detected in relation to hippocampal volume (Table 2). There was an association between higher body fat percentage larger hippocampal volume (Model 2, β: 6.21 [95% CI: 0.31, 12.11], *p*: 0.039, FDR corrected *p*: 0.10). However, this association did not remain after adjusting for multiple comparisons, and there was no evidence of an association in model 1 (Table 2). Higher handgrip strength was associated with larger hippocampal volume (Model 2, β: 2.00 [95% CI: 0.39, 3.61], *p*: 0.015, FDR corrected *p*: 0.044) (Table 3). Faster gait speed was associated with larger hippocampal volume, but the association did not remain after adjusting for multiple comparisons (Model 2, β: 174.61 [95% CI: 18.96, 330.27], *p*: 0.028, FDR corrected *p*: 0.084) (Table 3). No evidence of an association with hippocampal volume was detected when comparing participants with and without probable sarcopenia (Table 4).

### Linear regression β-Coefficients with 95% CI for associations of body composition measures, handgrip strength, and gait speed with predicted brain age estimated as the difference in years between chronological age and predicted biological age (BAG)

Higher muscle quantity (ALSTI) was associated with lower (younger) predicted brain age (Model 2, β: −0.31 [95% CI: −0.45, −0.16], *p*: <0.0001, FDR corrected *p*: 0.00013). Moreover, compared to participants with sarcopenic level muscle mass, those with normal level muscle mass had lower predicted brain age (Model 2, β: −0.72 [95% CI: −1.20, −0.24], *p*: 0.0031, FDR corrected *p*: 0.0082). There was an association between higher accumulation of VAT and higher predicted brain age that did not remain after adjusting for multiple comparisons (Model 2, β: 0.299 [95% CI: 0.031, 0.567], *p*: 0.029, FDR corrected *p*: 0.057). There was an association between higher body fat percentage and lower predicted brain age (Model 2, β: −0.035 [95% CI: −0.067, −0.002], *p*: 0.036, FDR corrected *p*: 0.058). However, this association did not remain after adjusting for multiple comparisons, and there was no evidence of an association in model 1 (Table 2). Evidence of associations between faster gait speed and lower predicted brain age (Model 1, β: −1.11 [95% CI: −1.91, −0.31], *p*: 0.0070), and between higher handgrip strength and lower predicted brain age (Model 1, β: −0.010 [95% CI: −0.019, −0.001], *p*: 0.022) were found in model 1, but the associations did not remain in model 2 (Model 2, β: −0.52 [95% CI: −1.38, 0.35], *p*: 0.24, FDR corrected *p*: 0.36), (Model 2, β: −0.008 [95% CI: −0.017, 0.001], *p*: 0.073, FDR corrected *p*: 0.15) (Table 3). Compared to participants with probable sarcopenia, those with no probable sarcopenia had lower predicted brain age (Model 2, β: −0.481 [95% CI: −0.869, −0.093], *p*: 0.015, FDR corrected *p*: 0.030) (Table 4).

### Ordinal logistic regression models with OR and 95% CI for associations of body composition measures, handgrip strength, and gait speed with CSVD burden (score range 0–3)

Compared to participants with sarcopenic level muscle mass, those with normal level muscle mass had lower CSVD burden (Model 2, OR: 0.58 [95% CI: 0.35, 0.97], *p*: 0.036, FDR corrected *p*: 0.064), and higher accumulation of VAT was associated with more CSVD burden (Model 2, OR: 1.41 [95% CI: 1.06, 1.88], *p*: 0.019, FDR corrected *p*: 0.071) (Table 2). However, these associations did not remain after adjusting for multiple comparisons. Faster gait speed was associated with lower CSVD burden (Model 2, OR: 0.31 [95% CI: 0.12, 0.77], *p*: 0.011, FDR corrected *p*: 0.043) (Table 3). No evidence of associations between either ALSTI, total body fat percentage, handgrip strength, or probable sarcopenia was detected in relation to CSVD burden (Table 2, Table 3, Table 4).

### Linear regression β-Coefficients with 95% CI for associations of body composition measures, handgrip strength, and gait speed with cognitive performance estimated with a global cognitive composite score

No evidence linking body composition with cognitive performance was detected (Table 2). Faster gait speed was associated with better cognitive performance (Model 2, β: 0.341 [95% CI: 0.065, 0.618], *p*: 0.016, FDR corrected *p*: 0.019), while no evidence of a linear association between handgrip strength and cognitive performance was found (Table 3). However, a link between probable sarcopenia and worse cognitive performance was detected when participants with no probable sarcopenia was compared to those with probable sarcopenia (Model 2, β: 0.181 [95% CI: 0.056, 0.305], *p*: 0.0045, FDR corrected *p*: 0.0053) (Table 4).

No evidence of sex-body composition interactions in relation to the MRI measures or cognitive performance was detected, except for an interaction between biological sex and total body fat percentage in relation to CSVD burden. However, no evidence of an association was found among either women (Model 2, OR: 0.98 [95% CI: 0.94, 1.03], *p*: 0.39), or men (Model 2, OR: 1.03 [95% CI: 0.97, 1.10], *p*: 0.36). Results from the analyses performed stratified by sex can be found in the Supplementary Material (Supplementary Tables S1–S3).

## Discussion

In this cross-sectional study of dementia-free 70-year-olds, we found associations of body composition with indicators of brain integrity and cognitive performance. Specifically, we found a linear association between higher muscle quantity and lower (younger) predicted brain age, and that those with normal level muscle mass had overall thicker cortex, thicker cortex in AD-signature regions, larger hippocampal volume, and lower predicted brain age compared to those with sarcopenic level muscle mass. Moreover, we found that probable sarcopenia was associated with higher (older) predicted brain age and worse cognitive performance, that higher hand grip strength was associated with larger hippocampal volume, and that faster gait speed was associated with less CSVD burden and better cognitive performance. Furthermore, we found that higher accumulation of VAT, but not higher total body fat percentage, was associated with overall thinner cortex.

Results from this study indicate that muscle mass at a sarcopenic level may be associated with lower brain integrity when examined in relation to measures of cortical thickness, hippocampal volume, and predicted brain age. Furthermore, the linear association between higher muscle mass and lower predicted brain age, suggests that age-related body-brain links may be detectable even before muscle mass reaches a sarcopenic level. Previous studies have found either no associations, or cross-sectional associations between higher lean mass and larger whole brain, grey matter, and white matter volumes.^12^^,^^13^ However, in one study where the participants were followed longitudinally, the associations did not remain over time.^13^ The authors hypothesised that differences between the cross-sectional and longitudinal results could be explained by the fact that the participants with a follow-up MRI scan were younger, higher educated, and healthier compared to those with only baseline MRI.^13^ However, as in our study, cross-sectional associations may also reflect cumulative risk or shared aetiology. Whether the associations of sarcopenic level muscle mass with thinner cortex and smaller hippocampal volume may be related to preclinical AD is difficult to determine in our study, as sarcopenic level muscle mass was associated with both overall thinner cortex and thinner cortex in AD-signature regions. Moreover, even though hippocampal atrophy is considered an early sign of AD due to its critical role in learning and memory, hippocampal atrophy can also be seen in normal ageing.^33^

We chose to explore the impact of muscle mass, muscle strength and function independently as the prevalence of sarcopenia was low in our sample, as reported previously.^20^ Furthermore, it has been suggested that mechanistic pathways linking sarcopenia with cognitive function may differ between components of sarcopenia.^6^ The exact mechanism linking muscle mass with brain structures and cognitive function is not known, but it has been suggested that there may be shared pathophysiological pathways involving factors such as mitochondrial dysfunction, inflammation, metabolic alterations, and neurological factors.^7^ It has also been suggested that skeletal muscle produces and secretes molecules, called myokines, that regulate brain functions, including mood, learning, and neuronal injury protection.^34^ Similar to previous studies, we found that higher muscle strength was associated with larger hippocampal volume, and with lower predicted brain age.^35^^,^^36^ The associations found in our study between both higher muscle mass and muscle strength (above sarcopenic levels) and lower predicted brain age indicate a link between early signs of sarcopenia and biological brain age. Thus, hypothetically, early interventions to reduce the risk of sarcopenia may be beneficial also for brain health. However, as in all cross-sectional studies, reversed causality, or bidirectional associations cannot be ruled out. Potentially, impaired brain health may reduce daily functioning and lead to a sedentary lifestyle, which in turn could contribute to the loss of muscle mass and function.^37^ Alternatively, shared pathways may lead to impairments in both brain and physical health.^38^ Thus, a decline in muscle mass and function could indicate brain health issues, even if the associations arise from cumulative risk, shared aetiology, or reversed causality. Early detection may offer opportunities to reverse conditions linked to body composition and brain health.^39^ Additionally, we found that better physical performance measured with gait speed was associated with less CSVD burden and better cognitive performance, which confirms results from previous studies.^11^^,^^40^^,^^41^

Obesity in midlife is considered a risk factor for dementia. Among older adults, a higher BMI has been associated with both increased and decreased dementia risk.^42^ However, rather than being a risk factor for dementia, a low BMI could be a consequence of weight loss (resulting in lower BMI) caused by the disease progression in the preclinical phase.^43^ Another partial explanation for this so called “obesity paradox” could be that BMI is a crude anthropometric measure that does not consider the distribution of body fat and muscle mass. VAT is considered an independent risk factor for cardiovascular and metabolic morbidity due to the secretion of adipocytokines and other vasoactive substances.^44^ The overall results from our study support the notion that VAT may be more detrimental than fat distributed in other body regions.^8^ In our study, we found that VAT was associated with overall thinner cortex. Furthermore, we found results indicating potential links between higher accumulation of VAT and more CSVD burden, thinner cortex in AD-signature cortical regions, and higher predicted brain age. We also found links between higher body fat percentage and overall thicker cortex, larger hippocampal volume and lower predicted brain age. However, these results should be interpreted with caution, as only the association between higher accumulation of VAT and overall thinner cortex remained after correcting for multiple comparisons. Furthermore, the associations with total body fat percentage were not seen in model 1, indicating an interplay with the potential confounders. Few other studies have explored these associations among older adults. However, a previous study found a U-shaped association between VAT and global cortical thickness.^16^ A Japanese study among older adults, found that higher accumulation of VAT was associated with more CSVD burden.^14^ Another study linked higher accumulation of VAT with higher predicted brain age, but found no links between body fat percentage and predicted brain age.^17^

Due to biological differences in body composition between women and men, sex-body composition interactions were explored in relation to the MRI measures and cognitive performance. No evident interactions were detected, with the exception of an interaction between total body fat percentage and sex in relation to CSVD burden. Potentially, this interaction could be explained by the fact that it is more common for men to accumulate fat around the trunk and abdomen, while women often accumulate fat around the hips and thighs, thus having less cardiometabolic risk compared to men. However, no evidence of an association between body fat percentage and CSVD were detected among either women or men. The overall lack of evidence for sex-body composition interactions in this study may be due to the fact that sex was considered when constructing the variables (e.g., different cutoffs for men and women), and because the interplay between body composition measures were considered when performing the analyses. Performing the analyses stratified by sex showed similar results in men and women, but the results indicate a loss of statistical power when the data was split by sex.

We found no evidence linking body composition with cognitive performance. Potentially, we may have found associations between body composition and brain structures at a stage where it has not yet influenced cognitive performance. Previous studies have shown a strong relationship between gait speed and cognitive performance among community-dwelling older adults.^11^ Also in our study, faster gait speed was associated with better cognitive performance. Furthermore, even though no evidence of a linear association between handgrip strength and cognitive performance was detected, we found that probable sarcopenia (estimated by hand grip strength cutoffs) was associated with worse cognitive performance. These results indicate that markers of physical performance may be stronger/earlier indicators of cognitive decline than body composition among dementia-free older adults. Overall, our results support the notion that improving muscle fitness could be beneficial for brain health.

### Strengths and limitations

Strengths of this study were the systematically selected well-characterised community-based sample, and the comprehensive medical, behavioural, and cognitive examinations performed by trained staff. The systematic selection of study participants helped minimise selection bias related to recruitment, which increased the possibility to achieve a representative sample. Other strengths were the wide-ranging brain MRI markers included, and the use of DXA to measure body composition.

Some limitations need to be addressed. First, the cross-sectional design limits causal interpretation, as it cannot determine the direction of associations or rule out the possibility of non-causal relationships driven by shared risk factors. Intervention studies are required to confirm whether targeting body composition promotes healthy brain ageing and reduces the risk of cognitive impairment. Second, as in any observational study, residual confounding cannot be completely ruled out. However, residual confounding of potential measurement errors may have been limited by the multidisciplinary approach of the study, including trained and specialised research staff performing the interviews, health examinations and diagnostics. Third, although the response rate was high in this study, it is possible that participants may differ from refusals and the general population regarding potentially important factors. Fourth, potential incidence-prevalence bias may have occurred as we estimated body-brain links on a given time point in participants that have survived to the age of 70 years. Furthermore, the MRI sample were slightly healthier and higher educated than the non-MRI sample,^45^ potentially weakening the detection of an association. Fifth, performing multiple testing amplifies the probability of false-positive findings. However, FDR-adjusted *p*-values were calculated to address this issue. Sixth, additive interaction on the risk scale is preferred in terms of causality and public health. Using product term in the ordinal logistic model or comparing odds ratios is equivalent to multiplicative interaction assessment on the odds scale. We cannot rule out the possibility that absence of interaction on one scale may suggest presence of interaction on another scale. Finally, the proportion participants born outside Sweden was slightly lower in our study sample (15.5%) than among 70-year-olds in Gothenburg (19.5%), and not speaking Swedish was an exclusion criterium (n = 53 was excluded).^19^ Thus, the results may only be possible to generalise to populations with similar characteristics as the 70-year-olds examined in this study. However, the mechanistic insights could potentially be generalised also to other groups.

### Conclusions

In this cross-sectional study of dementia-free 70-year-olds, we found that low muscle mass and higher accumulation of visceral fat were associated with indicators of lower brain integrity, as measured by neuroimaging biomarkers, while no evidence of associations with total body fat percentage was detected. Moreover, the results support the notion that better physical performance is associated with better cognitive performance. Overall, the results indicate that improving muscle mass fitness and lower visceral fat may be beneficial for brain health. The insights from this study can be useful for future studies exploring links between body composition and dementia risk, and for preventive strategies aimed to promote healthy brain ageing, as factors related to body composition may be modifiable.

## Contributors

Conception and design of the study: Jessica Samuelsson and Ingmar Skoog. Acquisition of data: Ingmar Skoog, Ola Wallengren, Olof Lindberg, Sarah Shams, Caroline Dartora, Eric Westman. Funding acquisition: Ingmar Skoog. Methodology and interpretation of data: Jessica Samuelsson, Anna Marseglia, Ola Wallengren. Data analysis: Jessica Samuelsson. Drafting the article: Jessica Samuelsson. Revising the article critically for important intellectual content: Anna Marseglia, Ola Wallengren, Olof Lindberg, Caroline Dartora, Nira Cedrés, Sarah Shams, Silke Kern, Anna Zettergren, Eric Westman, Ingmar Skoog. Jessica Samuelsson and Ingmar Skoog have accessed and verified the underlying data reported in the manuscript, and were responsible for the decision to submit the manuscript. All authors had full access to all the data in the study and had final responsibility for the decision to submit for publication. All authors read and approved the manuscript.

## Data sharing statement

Deidentified data from this study will be made available upon request with investigator support, after approval of a proposal, and with a signed data access agreement. For data access, please contact the corresponding author at jessica.samuelsson@neuro.gu.se.

## Declaration of interests

The authors of this manuscript declares that there are no conflicts of interest.
