# Supplementary material for: Association of body composition with neuroimaging biomarkers and cognitive function; a population-based study of 70-year-olds
Source: eBioMedicine. 2025 Jan 8;112:105555. doi: 10.1016/j.ebiom.2024.105555 (PMC11762906; doi:10.1016/j.ebiom.2024.105555)
Supplement: Supplementary Figure and Tables [file mmc1.docx]

**Supplemental data**

**Table of contents:**

| Supplementary figure 1 | Causal directed acyclic graph (DAG) to identify confounders……………………... | 2 |
| --- | --- | --- |
| Supplementary table 1 | Linear regression β-Coefficients or ordinal logistic regression OR with 95% confidence intervals for associations of body composition measures with neuroimaging biomarkers and cognitive function among women and men………………………………………………………………………………….. | 3-4 |
| Supplementary table 2 | Linear regression β-Coefficients or ordinal logistic regression OR with 95% confidence intervals for associations of hand grip strength and gait speed with neuroimaging biomarkers and cognitive function among women and men……………………………………………………....................................... | 5 |
| Supplementary table 3 | Linear regression β-Coefficients or ordinal logistic regression OR with 95% confidence intervals for associations of probable sarcopenia with neuroimaging biomarkers and cognitive function among women and men………………………….………………………………………………………. | 6 |

**
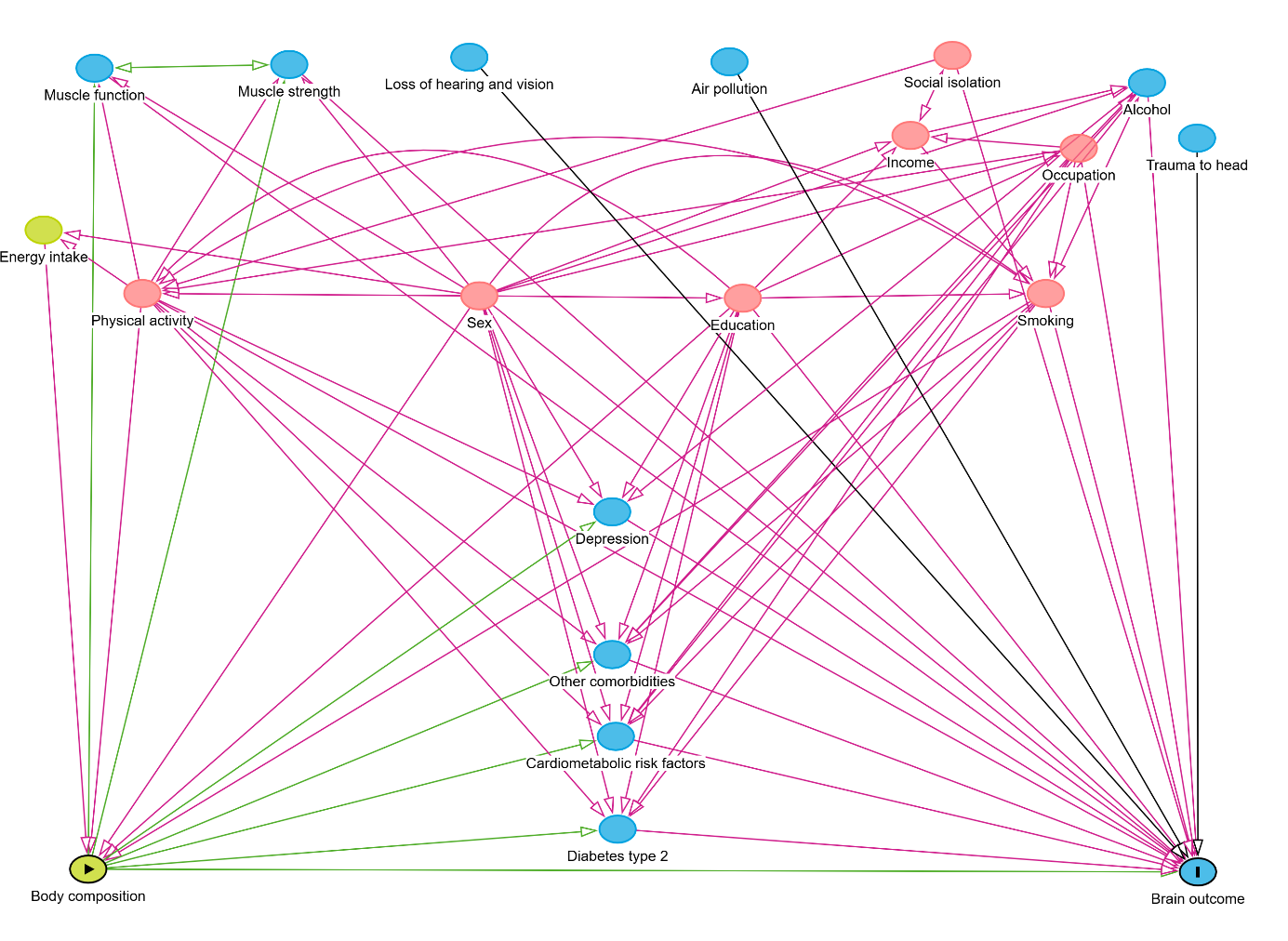
Supplementary Figure 1**. **A Directed Acyclic Graph (DAG) to explore potential confounders**. The selection of potential confounders was based on theoretical considerations. The DAG was constructed to focus on the primary relationships of interest, specifically the possible impact of body composition measures on brain outcome such as cortical thickness, hippocampal volume, predicted brain age, small vessel disease, and cognitive performance. The minimal sufficient adjustment set included smoking, physical activity, sex, and education.

**Supplementary table 1. Linear regression β-Coefficients or ordinal logistic regression OR with 95% confidence intervals for associations of body composition with neuroimaging biomarkers and cognitive function among women and men.**

| **N=674** | **Women N=362** |  |  | **Men N=312** |  |  |
| --- | --- | --- | --- | --- | --- | --- |
|  | **β (95% CI)** |  | ***P*-value** | **β (95% CI)** |  | ***P*-value** |
| **Total mean cortical thickness^ß^** |  |  |  |  |  |  |
| Appendicular lean soft tissue index (ALSTI)^¶^ | 0.004 (-0.005, 0.013) |  | 0.41 | 0.003 (-0.007, 0.012) |  | 0.58 |
| Normal level muscle mass compared to sarcopenic level muscle mass^¶^ | 0.051 (0.019, 0.083) |  | 0.0016 | 0.040 (0.012, 0.068) |  | 0.0056 |
| Total body fat percentage | 0.001 (-0.000, 0.003) |  | 0.16 | 0.002 (-0.001, 0.005) |  | 0.12 |
| Visceral adipose tissue | -0.018 (-0.037, 0.000) |  | 0.046 | -0.016 (-0.034, 0.002) |  | 0.087 |
| **AD-signature cortical thickness^ß^** |  |  |  |  |  |  |
| Appendicular lean soft tissue index (ALSTI)^¶^ | 0.005 (-0.006, 0.016) |  | 0.38 | 0.005 (-0.007, 0.017) |  | 0.43 |
| Normal level muscle mass compared to sarcopenic level muscle mass^¶^ | 0.059 (0.020, 0.098) |  | 0.0028 | 0.049 (0.014, 0.089) |  | 0.0063 |
| Total body fat percentage | 0.000 (-0.002, 0.002) |  | 0.71 | 0.003 (-0.000, 0.006) |  | 0.087 |
| Visceral adipose tissue | -0.014 (-0.036, 0.008) |  | 0.21 | -0.023 (-0.046, 0.000) |  | 0.047 |
| **Hippocampal volume^ß^** |  |  |  |  |  |  |
| Appendicular lean soft tissue index (ALSTI)^¶^ | 19.30 (-18.71, 57.31) |  | 0.32 | 27.33 (-12.58, 67.23) |  | 0.18 |
| Normal level muscle mass compared to sarcopenic level muscle mass^¶^ | 169.56 (38.99, 300.13) |  | 0.011 | 82.91 (-37.01, 202.83) |  | 0.18 |
| Total body fat percentage | 2.96 (-4.05, 9.97) |  | 0.41 | 13.13 (1.48, 24.77) |  | 0.027 |
| Visceral adipose tissue | -14.26 (87.76, 59.24) |  | 0.70 | -48.04 (-125.75, 29.67) |  | 0.23 |
| **Predicted brain age^*^** |  |  |  |  |  |  |
| Appendicular lean soft tissue index (ALSTI)^¶^ | -0.346 (-0.560, -0,131) |  | 0.0020 | -0.243 (-0.453, -0.033) |  | 0.023 |
| Normal level muscle mass compared to sarcopenic level muscle mass^¶^ | -0.499 (-1.249, 0.252) |  | 0.19 | -0.833 (-1.462, -0.203) |  | 0.010 |
| Total body fat percentage | -0.036 (-0.075, 0.004) |  | 0.074 | -0.011 (-0.073, 0.050) |  | 0.72 |
| Visceral adipose tissue | 0.339 (-0.076, 0.753) |  | 0.11 | 0.079 (-0.330, 0.488) |  | 0.71 |
| **Global cognitive composite score^µ^** |  |  |  |  |  |  |
| Appendicular lean soft tissue index (ALSTI)^¶^ | 0.059 (-0.009, 0.128) |  | 0.089 | -0.028 (-0.98, 0.042) |  | 0.43 |
| Normal level muscle mass compared to sarcopenic level muscle mass^¶^ | 0.206 (-0.031, 0.443) |  | 0.088 | -0.092 (-0.304, 0.119) |  | 0.39 |
| Total body fat percentage | -0.005 (-0.018, 0.008) |  | 0.44 | -0.013 (-0.034, 0.007) |  | 0.20 |
| Visceral adipose tissue | -0.103 (-0.235, 0.030) |  | 0.13 | 0.042 (-0.095, 0.179) |  | 0.55 |
|  | **OR**  **(95% CI)** |  | ***P*-value** | **OR**  **(95% CI)** |  | ***P*-value** |
| **Cerebral small vessel disease score^≈^** |  |  |  |  |  |  |
| Appendicular lean soft tissue index (ALSTI)^¶^ | 0.97 (0.76, 1.24) |  | 0.83 | 0.91 (0.73, 1.13) |  | 0.37 |
| Normal level muscle mass compared to sarcopenic level muscle mass^¶^ | 0.67 (0.29, 1.52) |  | 0.34 | 0.60 (0.32, 1.15) |  | 0.13 |
| Total body fat percentage | 0.98 (0.94, 1.03) |  | 0.39 | 1.03 (0.97, 1.10) |  | 0.36 |
| Visceral adipose tissue | 1.28 (0.80, 2.04) |  | 0.31 | 1.20 (0.78, 1.86) |  | 0.41 |

Note: Analyses were performed with linear or ordinal logistic regression models. Dependent variables in the linear regression models were total mean cortical thickness, mean cortical thickness in AD-signature regions, hippocampal volume, predicted brain age, and a global cognitive composite score. Dependent variable in the ordinal logistic regression model (cumulative logit model) was the cerebral small vessel disease (CSVD) score. The independent variables ALSTI, total body fat percentage and Visceral adipose tissue (VAT) were investigated in the same model, adjusted for sex, physical activity, education, and smoking, with results reported separately for each body composition measure. ALSTI dichotomised (normal level muscle mass/sarcopenic level muscle mass) was investigated in a separate model adjusted for sex, VAT, body fat percentage, physical activity, education, and smoking. There were missing VAT data for 3 participants (0.4%), and missing CSVD data for 4 participants (0.5%).

**^¶^**ALSTI: Appendicular lean soft tissue index (kg/body height m^2^) was used as a marker of muscle mass. In the analyses, ALSTI (continuous) was adjusted for sex by using the residuals of a linear regression model with appendicular lean soft tissue in kg as the outcome variable, and interaction variables of sex and body height as predictors. Sarcopenic level muscle mass was classified as: below the EWGSOP2 sarcopenia cut-off of ALSTI <7.0 for men, and <5.5 for women, and normal level muscle mass was classified as: levels above the ALSTI cutoffs.

^ß^Total mean cortical thickness is a measured in mm. Alzheimer’s disease (AD)-signature: Averaging cortical thicknesses of entorhinal, inferior temporal, middle temporal, and fusiform regions, adjusted for surface area, measured in mm. Hippocampal volume was adjusted for total intracranial volume, measured in mm^3^.

**^µ^**The global cognitive composite score (z-score) was derived from eight cognitive tests covering five cognitive domains: executive function, perceptual speed, verbal fluency, episodic memory, and visuospatial abilities.

*Predicted brain age was investigated by the difference in years between chronological age and predicted biological age (brain age gap, BAG). A negative value indicates a biologically younger brain, and a positive value a biological older brain, compared to chronological age.

^≈^The CSVD score ranged from 0 (no cerebrovascular burden) – 3 (high cerebrovascular burden).

**Supplementary table 2. Linear regression β-Coefficients or ordinal logistic regression OR with 95% confidence intervals for associations of hand grip strength and gait speed with neuroimaging biomarkers and cognitive function among women and men.**

| **N=674** | **Women N=362** | |  |  | **Men N=312** | |  |  |
| --- | --- | --- | --- | --- | --- | --- | --- | --- |
|  | **β (95% CI)** | |  | ***P*-value** | **β (95% CI)** | |  | ***P*-value** |
| **Total mean cortical thickness^ß^** |  | |  |  |  | |  |  |
| Hand grip strength | 0.0002 (-0.0003, 0.0007) | |  | 0.44 | 0.0001 (-0.0004, 0.0006) | |  | 0.70 |
| Gait speed | 0.015 (-0.034, 0.064) | |  | 0.55 | 0.061 (0.004, 0.118) | |  | 0.038 |
| **AD-signature cortical thickness^ß^** |  | |  |  |  | |  |  |
| Hand grip strength | 0.0001 (-0.0004, 0.0008) | |  | 0.65 | 0.0003 (-0.0003, 0.0010) | |  | 0.39 |
| Gait speed | 0.027 (-0.032, 0.086) | |  | 0.36 | 0.063 (-0.009, 0.135) | |  | 0.086 |
| **Hippocampal volume^ß^** |  | |  |  |  | |  |  |
| Hand grip strength | 2.22 (0.058, 4.38) | |  | 0.044 | 1.69 (-0.71, 4.08) | |  | 0.17 |
| Gait speed | 134.38 (-65.27, 334.03) | |  | 0.198 | 228.25 (-14.99, 471.48) | |  | 0.066 |
| **Predicted brain age^*^** |  | |  |  |  | |  |  |
| Hand grip strength | -0.008 (-0.020, 0.005) | |  | 0.22 | -0.008 (-0.020, 0.005) | |  | 0.30 |
| Gait speed | 0.055 (-1.092, 1.202) | |  | 0.93 | -0.981 (-2.257, 0.295) | |  | 0.13 |
| **Global cognitive composite score^µ^** |  | |  |  |  | |  |  |
| Hand grip strength | 0.000 (-0.004, 0.004) | |  | 0.97 | 0.002 (-0.002, 0.007) | |  | 0.27 |
| Gait speed | 0.391 (0.030, 0.751) | |  | 0.034 | 0.298 (-0.129, 0.725) | |  | 0.17 |
|  | **OR**  **(95% CI)** | |  | ***P*-value** | **OR**  **(95% CI)** | |  | ***P*-value** |
| **Cerebral small vessel disease score ^≈^** |  | |  |  |  |  | |  |
| Hand grip strength | 0.99 (0.98, 1.01) |  | | 0.44 | 0.99 (0.98, 1.01) |  | | 0.45 |
| Gait speed | 0.30 (0.08, 1.13) |  | | 0.076 | 0.28 (0.08, 1.01) |  | | 0.052 |

Note: Analyses were performed with linear or ordinal logistic regression models. Dependent variables in the linear regression models were total mean cortical thickness, mean cortical thickness in AD-signature regions, hippocampal volume, predicted brain age, and a global cognitive composite score. Dependent variable in the ordinal logistic regression model (cumulative logit model) was the cerebral small vessel disease (CSVD) score. Independent variables were hand grip strength and gait speed. Hand grip strength and gait speed were investigated in separate analyses. The models were adjusted for sex, physical activity, education, and smoking. There were missing CSVD data for 4 participants, missing gait speed data for 10 participants (1.4%), and missing hand grip strength data for 1 participant (0.1%).

**^§^**Handgrip strength (kPa), best value out of three on the dominant hand.

**^£^**Gait speed self-selected 30 meter indoor (meter/second).

^ß^Total mean cortical thickness is measured in mm. Alzheimer’s disease (AD)-signature: Averaging cortical thicknesses of entorhinal, inferior temporal, middle temporal, and fusiform regions, adjusted for surface area, measured in mm. Hippocampal volume was adjusted for total intracranial volume, measured in mm^3^.

**^µ^**The global cognitive composite score (z-score) was derived from eight cognitive tests covering five cognitive domains: executive function, perceptual speed, verbal fluency, episodic memory, and visuospatial abilities.

*Predicted brain age is investigated by the difference in years between chronological age and predicted biological age (brain age gap). A negative value indicates a biologically younger brain, and a positive value a biological older brain, compared to chronological age.

^≈^The CSVD score ranged from 0 (no cerebrovascular burden) – 3 (high cerebrovascular burden).

**Supplementary table 3. Linear regression β-Coefficients or ordinal logistic regression OR with 95% confidence intervals for associations of probable sarcopenia with neuroimaging biomarkers and cognitive function among women and men.**

| **N=674** | **Women N=362** |  |  | **Men N=312** |  |  |
| --- | --- | --- | --- | --- | --- | --- |
|  | **β (95% CI)** |  | ***P*-value** | **β (95% CI)** |  | ***P*-value** |
| **Total mean cortical thickness^ß^** |  |  |  |  |  |  |
| No probable sarcopenia compared to probable sarcopenia^¶^ | 0.004 (-0.017, 0.026) |  | 0.69 | -0.003 (-0.030, 0.024) |  | 0.82 |
| **AD-signature cortical thickness^ß^** |  |  |  |  |  |  |
| No probable sarcopenia compared to probable sarcopenia^¶^ | 0.002 (-0.024, 0.028) |  | 0.88 | -0.008 (-0.042, 0.025) |  | 0.63 |
| **Hippocampal volume^ß^** |  |  |  |  |  |  |
| No probable sarcopenia compared to probable sarcopenia^¶^ | 17.62 (-69.89, 105.23) |  | 0.69 | 33.52 (-79.75, 146.79) |  | 0.56 |
| **Predicted brain age^*^** |  |  |  |  |  |  |
| No probable sarcopenia compared to probable sarcopenia^¶^ | -0.406 (-0.907, 0.095) |  | 0.11 | -0.525 (-1.117, 0.067) |  | 0.082 |
| **Global cognitive composite score^µ^** |  |  |  |  |  |  |
| No probable sarcopenia compared to probable sarcopenia^¶^ | 0.132 (-0.026, 0.290) |  | 0.10 | 0.230 (0.033, 0.427) |  | 0.022 |
|  | **OR**  **(95% CI)** |  | ***P*-value** | **OR**  **(95% CI)** |  | ***P*-value** |
| **Cerebral small vessel disease score ^≈^** |  |  |  |  |  |  |
| No probable sarcopenia compared to probable sarcopenia^¶^ | 1.00 (0.58, 1.74) |  | 0.99 | 0.72 (0.39, 1.34) |  | 0.30 |

Note: Analyses were performed with linear or ordinal logistic regression models. Dependent variables in the linear regression models were total mean cortical thickness, mean cortical thickness in AD-signature regions, hippocampal volume, predicted brain age, and a global cognitive composite score. Dependent variable in the ordinal logistic regression model (cumulative logit model) was the cerebral small vessel disease (CSVD) score. Independent variable was probable sarcopenia (yes/no). The models were adjusted for sex, physical activity, education, and smoking. There were missing CSVD data for 4 participants, missing gait speed data for 10 participants (1.4%), and missing hand grip strength data for 1 participant (0.1%).

^¶^ Handgrip strength (kPa), best value out of three on the dominant hand. Probable sarcopenia was categorised based on handgrip strength below cutoff: <59 kPa for women and <69 kPa for men.

^ß^Total mean cortical thickness is measured in mm. Alzheimer’s disease (AD)-signature: Averaging cortical thicknesses of entorhinal, inferior temporal, middle temporal, and fusiform regions, adjusted for surface area, measured in mm. Hippocampal volume was adjusted for total intracranial volume, measured in mm^3^.

**^µ^**The global cognitive composite score (z-score) was derived from eight cognitive tests covering five cognitive domains: executive function, perceptual speed, verbal fluency, episodic memory, and visuospatial abilities.

*Predicted brain age is investigated by the difference in years between chronological age and predicted biological age (brain age gap). A negative value indicates a biologically younger brain, and a positive value a biological older brain, compared to chronological age.

^≈^The CSVD score ranged from 0 (no cerebrovascular burden) – 3 (high cerebrovascular burden).
